# Plasmonic Biosensor on the End-Facet of a Dual-Core Single-Mode Optical Fiber

**DOI:** 10.3390/bios13050558

**Published:** 2023-05-19

**Authors:** Fatemeh Fouladi Mahani, Arash Mokhtari, Pierre Berini

**Affiliations:** 1 Optical and RF Communication Systems (ORCS) Lab, Electrical Engineering Department, Shahid Bahonar University of Kerman, Kerman 7616913439, Iran; 2 Department of Physics, School of Electrical Engineering and Computer Science, University of Ottawa, Ottawa, ON K1N 6N5, Canada

**Keywords:** biosensors, dual-core optical fibers, lab-on-a-fiber, plasmonics

## Abstract

Optical biosensors target widespread applications, such as drug discovery, medical diagnostics, food quality control, and environmental monitoring. Here, we propose a novel plasmonic biosensor on the end-facet of a dual-core single-mode optical fiber. The concept uses slanted metal gratings on each core, interconnected by a metal stripe biosensing waveguide to couple the cores via the propagation of surface plasmons along the end facet. The scheme enables operation in transmission (core-to-core), thereby eliminating the need to separate the reflected light from the incident light. Importantly, this simplifies and reduces the cost of the interrogation setup because a broadband polarization-maintaining optical fiber coupler or circulator is not required. The proposed biosensor enables remote sensing because the interrogation optoelectronics can be located remotely. In vivo biosensing and brain studies are also enabled because the end-facet can be inserted into a living body, once properly packaged. It can also be dipped into a vial, precluding the need for microfluidic channels or pumps. Bulk sensitivities of 880 nm/RIU and surface sensitivities of 1 nm/nm are predicted under spectral interrogation using cross-correlation analysis. The configuration is embodied by robust and experimentally realizable designs that can be fabricated, e.g., using metal evaporation and focused ion beam milling.

## 1. Introduction

Optical fiber biosensors have found widespread application in many fields, such as drug discovery, medical diagnostics, food quality control, environmental monitoring, and agriculture [1]. Increasing attention has been drawn towards revolutionizing health care by designing biosensors with robust, fast, and real-time detection capabilities [2]. Exploiting the high surface sensitivity of surface plasmons on planar or resonant nanostructures to sense the binding interaction between a bio-specific pair has enabled many biosensing applications [3]. The most common plasmonic approach uses a Au film deposited on a prism (Kretschmann–Raether configuration) in contact with the sensing fluid, where the angle of surface plasmon excitation is monitored by measuring a dip in the reflectance—such systems are widely referred to as surface plasmon resonance (SPR) systems [4]. Alternatively, the use of optical fibers enables lab-on-a-fiber (LOF) platforms, which benefit from the superior waveguiding ability of optical fibers, allowing optoelectronic interrogation systems to be efficiently coupled and located remotely [5]. In this regard, various optical-fiber-based sensors have been presented by integrating different functional nanostructures within or around the holey structures of microstructured optical fibers [6], on the optical fiber’s outer curved surfaces (e.g., by means of tapered/unclad fibers [7] or diffraction gratings inscribed in the fiber core [8]), or on the tip of optical fibers [9]. Integrating plasmonic nanostructures on the tip (end-facet) of an optical fiber enables highly sensitive label-free biosensors that are robust, lightweight, and miniaturized [10].

Biosensors on the tip of an optical fiber usually operate in reflection [11]. For instance, in vivo monitoring of target analytes requires the insertion of the fiber end-facet into a living organism (as in an endoscope) such that the transmitted light is not accessible, which implies the need for interrogation in reflection [12]. Consequently, separating the reflected light from the incident light at the input is required. Furthermore, in the case of spectral interrogation, a tunable laser with a power sensor or a broadband optical source with a spectrometer is needed. Thus, separation of the reflected light from the incident light over a broad spectral range, usually while preserving polarization, is required. This is not trivial, as it requires the interrogation system to include high-cost ancillary optical components, such as a broadband polarization-maintaining optical fiber coupler or circulator.

A dual-core single-mode optical fiber (DCSMF), with a biosensor structure formed on its end-facet that also couples the cores, could overcome this challenge [13]. Employing a DCSMF facilitates optical interrogation as one core can be used to launch the incident light to the fiber tip, and the other core can be used to receive the light emerging from the biosensor. Such a system operates in transmission, which is advantageous, as it simplifies the interrogation setup, especially for polarization-resolved spectral interrogation. The idea was explored in a previous work, where the biosensor consisted of a Si_3_N_4_ grating-coupled Fabry–Pérot waveguide resonator, in which spectral interrogation in transmission was verified, but the design produced a bulk sensitivity of only 50–100 nm/RIU [13].

Here, we exploit surface plasmon polaritons (SPPs) in a high-sensitivity biosensor located on the end-facet of a DCSMF. This configuration requires coupling of normally incident light from the first core of the fiber to SPPs propagating along a metal stripe sensing waveguide connected to the second core ~40 µm away, then coupling SPPs to normally propagating light along the second core. Both couplers should be unidirectional, if possible, to improve the power efficiency. Furthermore, high sensitivity requires that the SPPs interact with the analyte, which implies that they should be excited and localized along the top metal surface, i.e., along the metal–analyte interface.

The challenge of efficient out-of-plane unidirectional coupling to SPPs has stimulated extensive studies. The structures used can be categorized mainly into slits [14], grooves [15], slit–grooves [16], ridge (grating) couplers [17], Bragg mirrors [18], and nano-antennas [19]. Unidirectional coupling requires that, for example, grating couplers have an asymmetric profile for perpendicular illumination [20,21].

In this paper, we propose a novel high-sensitivity plasmonic biosensor on the end-facet of a DCSMF. The structure comprises slanted metal gratings for unidirectional coupling and a metal sensing waveguide interconnecting the cores. Section 2 describes the concept as well as the proposed interrogation setup. Section 3 explains the design approach on the basis of intuitive vector geometry accompanied by modal calculations to attain the design parameters. Section 4 discusses the prospective fabrication steps and the design robustness relative to fabrication imperfections. Section 5 presents sensitivity computations, relying on cross-correlation analysis of the computed transmittance curves, aiming to evaluate the behavior of the biosensor. Concluding remarks are provided in Section 6.

## 2. Sensor Structure and Proposed Interrogation Setup

**Figure 1:**
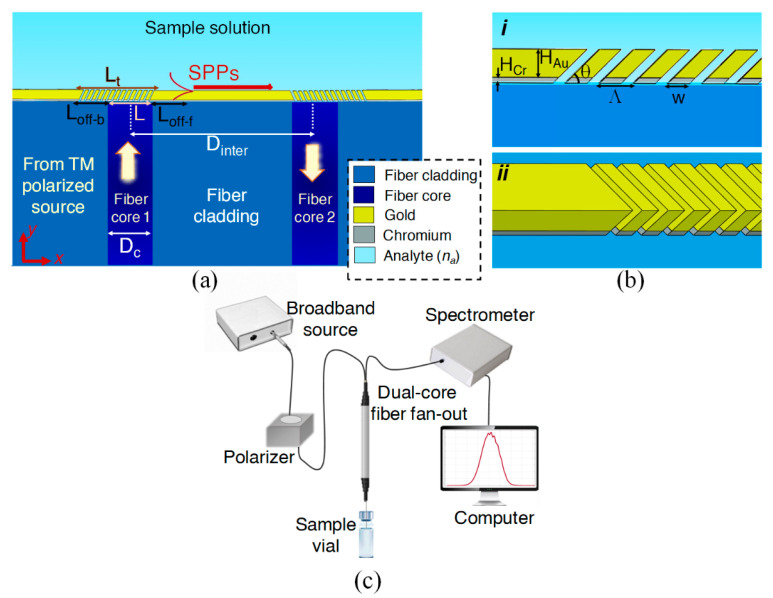
(**a**) Cross-sectional view of the plasmonic biosensor based on Au/Cr plasmonic slanted grating couplers (PSGCs) interconnected by a Au/Cr biosensing waveguide on the end-facet of a dual-core single-mode fiber (DCSMF). (**b**) Expanded views of the input PSGC in (***i***) longitudinal cross-sectional and (***ii***) 3D views. (**c**) Schematic diagram of a proposed experimental setup for interrogating the proposed biosensor.

A cross-sectional view of the proposed biosensor on the end-facet of a DCSMF is illustrated in Figure 1a, showing Au/Cr plasmonic slanted grating couplers (PSGCs) aligned with the cores, interconnected by a Au/Cr biosensing waveguide. Figure 1b provides sketches in expanded views of the input PSGC, defining important design dimensions. Transverse magnetic (TM)-polarized light is coupled to the input of the first core, emerging on the first PSGC, which couples the light to SPPs propagating along the top surface of the biosensing waveguide. The second PSGC then couples the SPPs into light propagating along the second core, emerging therefrom for detection. Au is used as the main material for the biosensor design since it is stable and easy to functionalize for a target biosensing application. Using a relatively thick Cr layer at the bottom of the Au layer promotes adhesion to the end-facet while eliminating undesired SPPs that may couple and propagate along the bottom of the biosensing waveguide [22].

Operating with a single-mode optical fiber is advantageous over a multi-mode one since the field distribution in the latter is much more complex and very sensitive to even weak external perturbations, such as displacement [23]. Here, the dimensions of a commercial DCSMF are assumed, for which low-loss fiber fan-outs are available [24]. The biosensor is designed to work at the operating wavelength of λ_0_ = 1.31 μm, for which high-quality and low-cost optoelectronics are available and because the absorption of water is small at this wavelength (biosensing fluids are mostly aqueous in nature) [25].

Although our design remains theoretical, to better understand the sensor operation, we show a sketch of the proposed interrogation setup in Figure 1c. A broadband source can be employed to excite the first core. Light emerging from the source is transmitted through a polarizer, ensuring operation with TM-polarized light, and a spectrometer is employed to capture the output power of the second core in a transmission arrangement. A polarization-maintaining DCSMF could be used, if available, to further simplify the setup. The biosensing sample is assumed to be in a vial, and the DCSMF end-facet bearing the biosensor is dipped therein, precluding the need for microfluidic channels or pumps. Properly packaged, the scheme has the potential for in vivo biosensing or brain studies.

## 3. Theoretical Design

We use finite element method (FEM) simulations in 2D with boundary mode analysis (BMA) to analyze the device and accurately investigate the sensor behavior [26].

**Figure 2:**
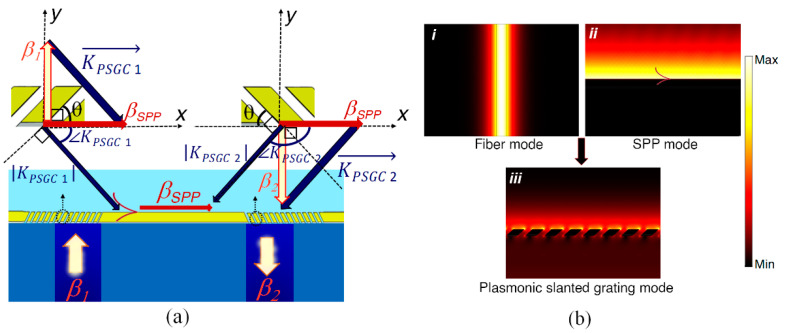
(**a**) Vector geometries and (**b**) TM_0_ mode field calculations for the theoretical design of the proposed plasmonic slanted grating couplers.

The main challenge in the design of the biosensor is achieving an appropriate scheme for unidirectional coupling of the input TM_0_ mode (propagation constant *β_1_*) in the first core to SPPs propagating along the top of the biosensing waveguide (propagation constant *β_SPP_*), followed by unidirectional coupling of the SPPs to the output TM_0_ mode (propagation constant *β_2_*) in the second core. The signal path is sketched in Figure 2a.

As mentioned earlier, the operating wavelength selected is 1.31 μm. The physical core/cladding dimensions of the optical fiber are set on the basis of a commercial DCSMF for which high-precision low-loss fiber fan-outs are available [24]. The fiber core and cladding diameters are 8 μm and 125 μm, respectively [24], and their refractive indices are 1.457 and 1.453, respectively, for single-mode operation near 1.31 μm. The length of the PSGCs should match the mode size of the fiber cores. So, very short PSGCs having a strong index modulation are required [27]. For this purpose, we propose using Au/Cr PSGCs comprising parallelogram-shaped ridges. To model the end-facet dipped into a sensing solution, we assume a refractive index (RI) of 1.333 for the region above the PSGCs and the biosensing waveguide (n_a_ = 1.333), which is typical for aqueous buffer solutions near our operating wavelength range. The sensing solution is assumed to infiltrate the slits of the PSGCs, ensuring a high material contrast with the metal ridges.

Contrary to low-contrast gratings for which the grating design can be determined from the phase-matching condition, the design of high-contrast grating couplers is more complex, as the coupling efficiency and (undesired) scattering into radiative modes depend on parameters such as slant angle, fill factor, and pitch. To overcome this challenge, we propose a straightforward but efficient design approach derived from an intuitive vector geometry accompanied by 1D modal calculations to attain initial design parameters, following a similar method adopted to design Si_3_N_4_ slanted gratings [13]. It will be shown that the initial design parameters are in good agreement with the optimized parameters obtained through numerical modeling.

Figure 2a outlines the proposed design steps. We design the PSGC on the first core, then use the same grating mirrored on the second core. The DCSMF is single-mode, so TM_0_-to-TM_0_ coupling between the cores is taken to define the core-to-core coupling efficiency. In the first step, we calculate the effective index and the propagation constant of the TM_0_ mode in the core by modeling the fiber as a slab waveguide, as shown in Part *i* of Figure 2b, yielding n_eff-1_ = 1.4548 and *β_1_* = 6.9778 × 10^6^ rad/m.

For the biosensing waveguide, we assume a 300 nm thick Au layer on a 50 nm Cr layer such that SPPs may propagate along the top Au–solution interface only (the bottom Cr-SiO_2_ interface does not effectively support SPPs) while also ensuring fabrication robustness, as discussed in the following section. The optical constants of Au and Cr are taken from experimental data [28,29]. The SPP mode on the top Au surface is also determined from modal analysis, as shown in Part *ii* of Figure 2b, resulting in *n_eff-SPP_* = 1.3482 − 0.0013821i and *β_SPP_* = 6.4664 × 10^6^ − 6629.1i rad/m. We use only the real parts of *n_eff-SPP_* and *β_SPP_* in our coupling estimates. The Au film is thick enough for the SPP mode to be taken as that of a single metal–dielectric interface, with its complex effective index given by the SPP dispersion formula [30]:

(1) $$n_{eff-SPP}=\sqrt{\frac{\varepsilon_{r,a}\varepsilon_{r,m}}{\varepsilon_{r,a}+\varepsilon_{r,m}}}$$

where *ε_r,a_* = n_a_^2^, and *ε_r,m_* is taken as the relative permittivity of Au. This formula yields the same result as the modal calculation described above.

As shown in the simple vector diagram of Figure 2a, the wavevector of the first PSGC, *K_PSGC_*_1_, which is perpendicular to the slanted ridges [31], can be approximated by the vector difference between the propagation constant of the TM_0_ mode in the fiber core and the propagation constant of the SPP excitation (*β_1_* and *β_SPP_*, respectively). Thus, using the ensuing geometric equations, the PSGC wavevector (*K_PSGC_*), slant angle (*θ*), propagation constant (*β_PSGC_*), and period (Λ) can be estimated:

(2) $$\left|K_{PSGC}\right|=\sqrt{{\left|\beta_{1}\right|}^{2}+{\left|\beta_{SPP}\right|}^{2}}$$

(3) $$\theta =\sin^{-1}(\frac{\beta_{SPP}}{\left|K_{PSGC}\right|})$$

(4) $$\beta_{PSGC}=K_{PSGC}\sin(\theta )$$

(5) $$\Lambda =\frac{2\pi }{\beta_{PSGC}}$$

(6) $$n_{PSGC}=\frac{\beta_{PSGC}}{k_{0}}$$

where the free-space wavenumber is *k*_0_ *=* 2π/λ_0_. The initial value of Λ is then scaled to place the coupling wavelength at 1.31 μm. The effective index of the PSGCs, *n_PSGC_*, is set equal to the real part of *n_eff-SPP_*, which is 1.3482 (i.e., *β_PSGC_ = β_SPP_*). Then, we use the extracted period and slant angle to calculate the effective index of the PSGC, aiming to obtain a physical SPP mode (with field enhancement mostly along the top side of the slanted ridges), having a real part of the effective index close to 1.3482 (Part *iii* of Figure 2b). To achieve this goal, we alter the fill factor (ff = w/Λ) and the gold thickness (H_Au_) of the PSGC, obtaining ff = 60% and H_Au_ = 300 nm for our design. Table 1 summarizes our design.

The design summarized in Table 1 is validated via 2D FEM-based BMA of the entire system. In doing so, we set up two ports, one excitation port at the input of the first core and one receiving port at the output of the second core. Two BMA steps at the ports accompanied by a frequency response analysis of the full structure are carried out. The distance between the centers of the two cores is taken as D_inter_ = 46.6 μm [24]. The gratings are offset slightly from the cores to decrease undesired coupling in the opposite direction (L_off-b_ > L_off-f_ in Figure 1a). Perfectly matched layers (PMLs) and scattering boundary conditions are employed to avoid spurious reflections from the outer boundaries of the simulation domain.

**Figure 3:**
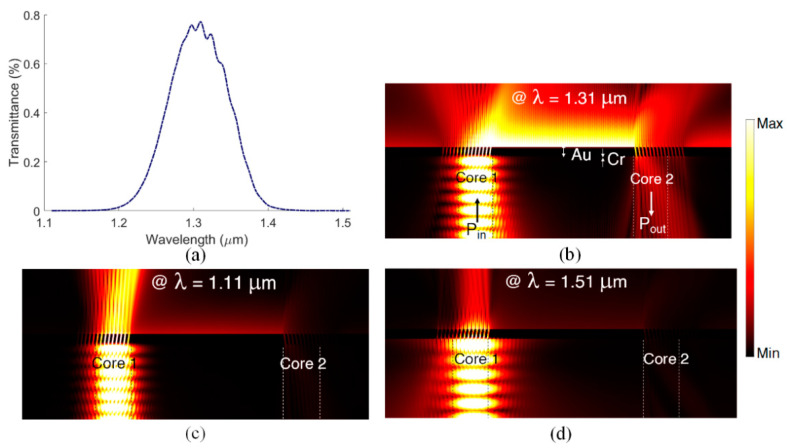
(**a**) Calculated core-to-core transmittance (TM_0_-to-TM_0_) from the first (P_in_) to the second (P_out_) core for the nominal design based on the vector geometry and modal calculations (Table 1). Normalized electric field distributions: (**b**) on-resonance; (**c**,**d**) off-resonance.

Figure 3a shows the transmittance of our design, defined as the ratio of power transmitted to the TM_0_ mode of the second core, P_out_, to the power carried by the input TM_0_ mode in the first core, P_in_. Despite the large distance between the two cores, the scheme provides a transmittance of up to −21.13 dB, ensuring that reasonable power will be received at the output. Directional grating coupling is evident from the computed on-resonance electric field profile of Figure 3b, especially when compared with the computed electric field profiles off-resonance, as shown in Figure 3c,d.

## 4. Proposed Fabrication Steps, Design Robustness, and Fabrication Tolerance

**Figure 4:**
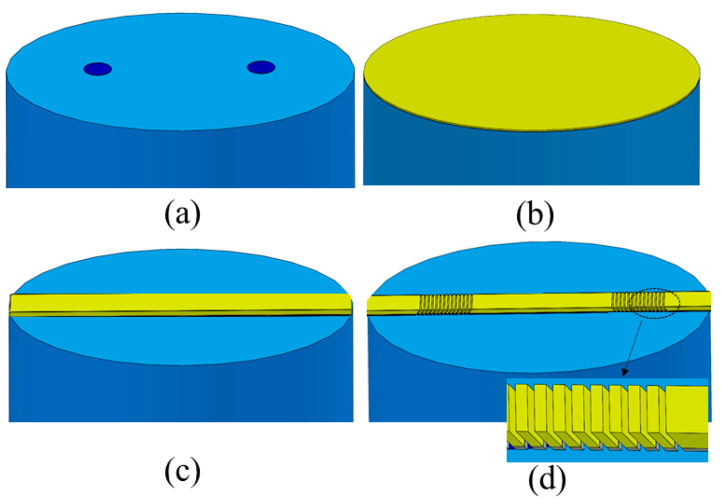
Proposed fabrication steps based on focused ion beam (FIB) milling. (**a**) Cleaved and polished end-facet of a DCSMF; (**b**) evaporation of Au and Cr layers; (**c**) FIB milling of excess metal to define the biosensing stripe waveguide; (**d**) FIB milling at an angle to define the slanted gratings.

Although our study is theoretical, we propose in Figure 4 steps to fabricate the biosensor, with justification based on the literature. As depicted in Figure 4a, the starting “substrate” consists of the cleaved and polished end-facet of a commercial DCSMF [24,32]. The fiber is threaded into a ferrule, mounted perpendicularly on a custom-machined jig or fixture with fiber management to enable mounting on vacuum tools, as in a previous work [33].

Then, a 50 nm thick Cr layer can be evaporated on the flat and smooth end-facet. This large thickness guarantees strong adhesion between the subsequent Au layer and the facet while also eliminating any undesired SPPs from being supported along the bottom surface of the metal film. Cr evaporation is subsequently followed by Au evaporation without breaking the vacuum, targeting a 300 nm thick Au layer, as shown in Figure 4b. The Au thickness selected ensures the long-term robustness of the sensing surface, and its resilience to chemical cleaning, facilitating multiple re-uses of the sensing surface (thick Au/Cr bi-layer or tri-layer stacks with near-perfect interfaces have successfully been realized in previous works [22,34]).

After cleaning and inspection, a focused ion beam (FIB) milling system can be used to mill the slanted gratings [35,36] and the biosensing waveguide directly on the end-facet, following previous works [33,37], as sketched in Figure 4c,d. Direct milling using a He ion beam has proven effective in forming delicate nanoscale features in a Au film [38]. For the slants, the facet can be tilted to mill at an angle. As discussed in the previous section, the designed slant angle (*θ*) is approximately 40°, and the grating pitch (Λ) and width (w) are approximately 980 nm and 580 nm, respectively, such that the slits are wider than the metal thickness, yielding a milled aspect ratio of less than 1. As discussed below, small changes in geometrical parameters due to the fabrication tolerance do not strongly affect the sensor response (e.g., *θ* = 60° yields almost the same result as *θ* = 40°).

**Figure 5:**
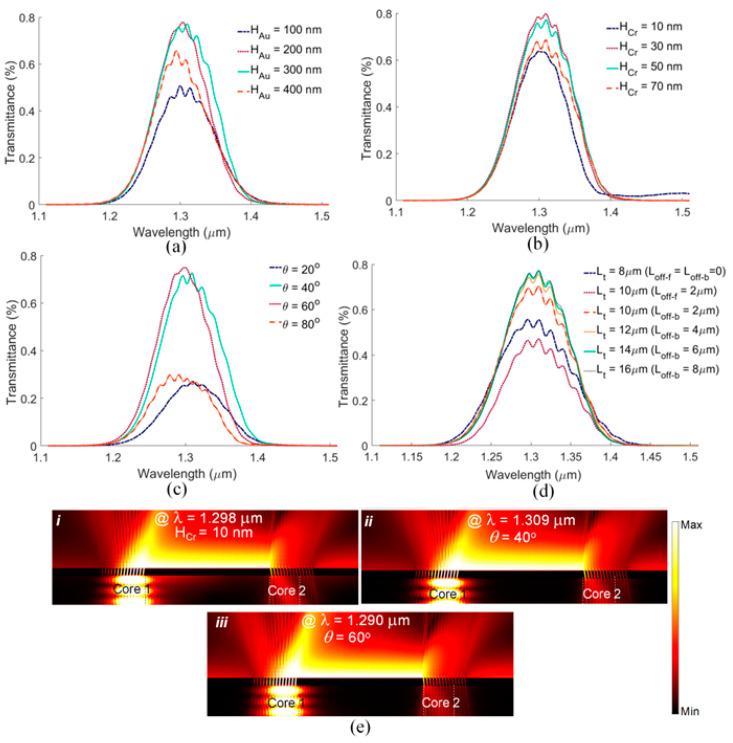
Fabrication tolerance of the sensor determined as variations in core-to-core transmittance (TM_0_-to-TM_0_) due to changes in (**a**) gold thickness (H_Au_), (**b**) chromium thickness (H_Cr_), (**c**) slant angle (*θ*), and (**d**) length (L) of the gratings. (**e**) (***i***) Normalized on-resonance electric field distribution of the nominal design with H_Cr_ = 10 nm, demonstrating the undesired excitation of SPPs along the bottom interface, and (***ii***) normalized on-resonance electric field distribution of the nominal design with *θ* = 40° and (***iii***) *θ* = 60°, showing good robustness against small changes in FIB milling angle.

The influence on the transmittance due to changes in the nominal design of our grating (Table 1) is demonstrated in Figure 5, maintaining an RI of 1.333 for the sensing solution. Figure 5a shows the effect of changing the Au thickness on the optical response. A thick Au layer is needed to ensure efficient coupling by the PSGCs to the SPP mode propagating along the top Au surface. As seen, a 300 nm thick Au film provides good coupling while simultaneously offering robustness (due to thickness). A thick Cr layer is also needed to suppress the SPP along the bottom Au surface while providing strong adhesion between the end-facet and the Au layer. Figure 5b shows the effect of changing the Cr thickness on the sensor response. As shown in the electric field profile of Figure 5e Part *i*, a 10 nm Cr layer is not thick enough to completely suppress the undesired SPP propagating along the bottom. Therefore, we choose 50 nm for this layer to achieve better suppression. (Alternatively, a 200 nm Au layer on a 30 nm Cr layer can also provide good coupling, while reducing the thicknesses, easing fabrication).

In practice, fabrication imperfections may cause small changes in the structural parameters of realized biosensor. As discussed in the following section, our sensor design is sufficiently robust such that small changes in the grating parameters do not significantly alter the sensor performance. As seen in Figure 5c, small changes in the slant angle (due to small changes in the FIB milling angle) do not significantly affect the sensor response; the transmittance is not significantly affected for PSGC slant angles from *θ* = 40° to 60° near nominal (*θ* = ~43°, Table 1). This can be appreciated by comparing the on-resonance field distributions of Figure 5e, Parts *ii* with *iii*, which show no appreciable difference.

In addition, small changes in the grating length do not influence the sensor response significantly, which also supports design robustness (Figure 5d). We know that the fiber mode field is slightly larger than the core diameter, as the mode decays exponentially into the cladding. Thus, the length of the PSGCs should be at least as large as 1/e of the fiber mode field diameter. To investigate the effect of PSGC length on the sensor transmittance, we define forward and backward offset lengths, L_off-f_ and L_off-b_, respectively, in Figure 1a, which are added to the core diameter, L. The total length of a PSGC is denoted as L_t_. As presented in Figure 5d, setting the grating length to the core diameter (L_t_ = L) does not lead to the best coupling. Adding grating ridges in the forward direction does not improve the sensor transmittance. However, adding grating ridges in the backward direction increases the sensor transmittance by increasing the directivity of SPP excitation in the forward direction. Increasing L_off-b_ beyond 6 μm does not further increase the sensor transmittance. Thus, we choose L_t_ = 14 μm for our design (L_t_ = L + L_off-b_, L = 8 μm, and L_off-b_ = 6 μm). However, as seen, even 2 μm changes in L_t_ do not cause a significant change in the sensor response.

**Figure 6:**
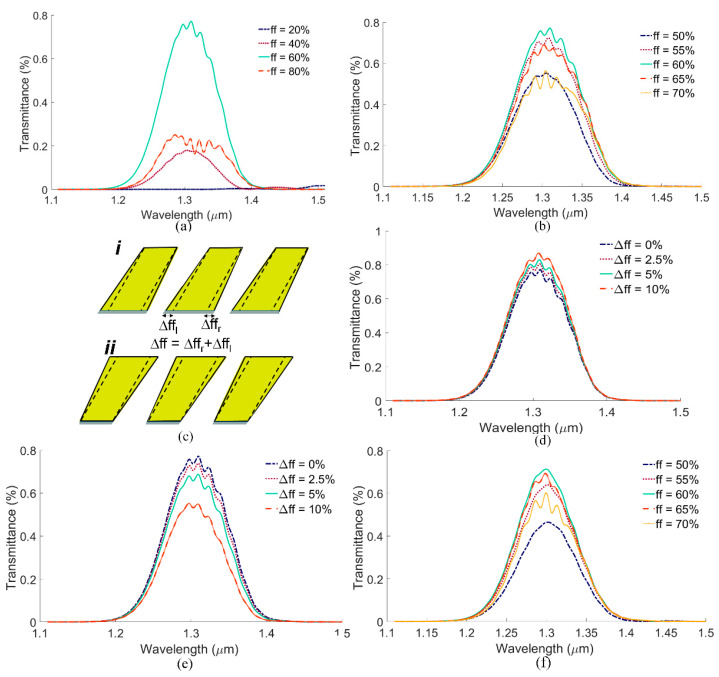
Fabrication tolerance of the sensor determined as variations in core-to-core transmittance (TM_0_-to-TM_0_) as the fill factor (ff) is changed in steps of (**a**) 20% and (**b**) 5%. (**c**) Slants of non-parallel sidewalls as (***i***) overcut or (***ii***) undercut features and their effect, (**d**) overcut and (**e**) undercut, on the sensor response. (**f**) Effect of changing fill factor (ff) in steps of 5% on the sensor design with H_Au_ = 200 nm, H_Cr_ = 30 nm, and *θ* = 45°.

The fill factor (ff) is also an important parameter, as it directly changes n_eff_ of the PSGC. As shown in Figure 6a, ff = 60% yields the best coupling, in agreement with our theoretical design (Table 1). For smaller or larger fill factors, the effective index of the PSGCs is altered such that the coupling efficiency is reduced. Moreover, ff = 60% presents a good aspect ratio from the fabrication viewpoint. As shown in Figure 6b, if ff changes slightly near 60% due to fabrication errors, the sensor response does not change significantly. Note that a 5% change in ff implies approximately 50 nm of change in the width (w) of the ridges, which corresponds to a large fabrication error.

Another fabrication error that might occur in FIB milling is the creation of slanted profiles with non-parallel sidewalls, having overcut or undercut slants [39], as shown in Figure 6c. The effect of overcut and undercut slants on the sensor response are depicted in Figure 6d,e, respectively. Here, the change in the ff, Δff, is defined as the summation of the right and left ff changes, Δff_r_ + Δff_l_. As seen, up to 5% change in the ff due to the overcutting and undercutting effects does not change the sensor response, also indicating sensor design robustness against fabrication errors.

As mentioned earlier, lower thicknesses of 200 nm for the Au layer and of 30 nm for the Cr layer reduce material costs and are also suitable for the sensor design. In addition, *θ* can be chosen as 45°, which may be easier to set practically. Figure 6f shows the effect of changing ff in steps of 5% on the sensor design, with H_Au_ = 200 nm, H_Cr_ = 30 nm, and *θ* = 45°, revealing almost the same robustness as the design involving larger thicknesses.

## 5. Sensitivity Computations

The conventional method for calculating the bulk sensitivity under wavelength interrogation is to determine the shift in the resonance wavelength (Δλ_r_) resulting from changes in the bulk RI of the sensing solution (Δn_a_), S = Δλ_r_/Δn_a_. Another important parameter of sensor performance is the figure of merit (FoM), defined as FoM = S/FWHM [40], where FWHM denotes the full-width at half-maximum of the transmittance spectrum.

**Figure 7:**
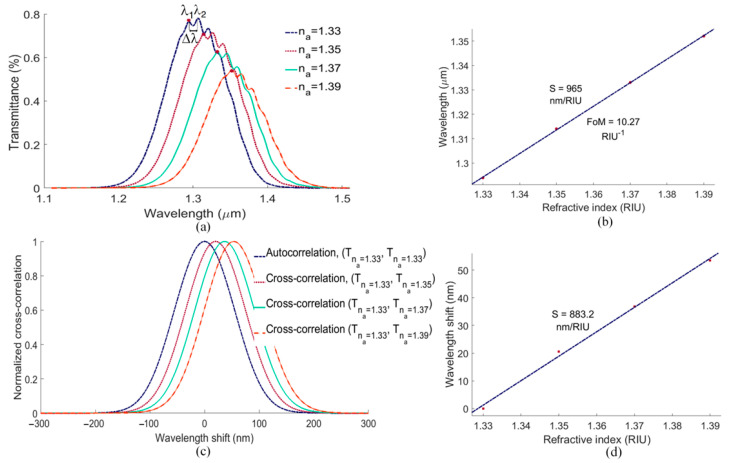
(**a**) The shift in the transmittance spectrum as the RI of the sensing solution is varied in the range from 1.33 to 1.39. (**b**) The shift in the wavelength of the feature marked by the solid red dot in Part (**a**) vs. the RI. The bulk sensitivity is taken as the slope of the best-fit linear model through the data. (**c**) Calculated normalized autocorrelation and cross-correlation of the transmittance responses of Part (**a**). (**d**) The shift in the peak cross-correlation vs. the RI. The bulk sensitivity is taken as the slope of the best-fit linear model through the data.

Figure 7a shows the sensor transmittance as the RI of the sensing solution varies from 1.33 to 1.39, a range typical for bio-compatible sensing solutions. As observed, changing n_a_ brings about not only obvious shifts in the transmittance but also slight changes in spectral shape and in the fringes comprising the spectra. These tiny fringes in the optical response of the structure originate from the weak Fabry–Pérot cavity formed between the mirrored PSGCs, as verified by calculating the distance between adjacent fringes (e.g., Δλ in Figure 7a), which is of the order of the free spectral range (FSR) for such a resonator [13]. Choosing one of the fringes, e.g., the first clear fringe in each spectrum, marked by the solid red dot in Figure 7a, and tracking its wavelength shift vs. n_a_ yields the plot of Figure 7b, from which a bulk sensitivity and FoM of 965 nm/RIU and 10 RIU^−1^, respectively, are deduced.

However, if the spectral shape evolves, a monitoring approach based on identifying and tracking a single feature in a complex spectral response may not be robust. Alternatively, we propose the calculation and tracking of the cross-correlation between transmittance curves. For this purpose, we choose one of the curves as our reference, e.g., the transmittance for n_a_ = 1.33 (T_na_ = 1.33), and calculate the normalized autocorrelation of this reference spectrum and the normalized cross-correlation between all other transmittance curves and T_na_ = 1.33, as plotted in Figure 7c. We then plot the shift in the cross-correlated curves vs. n_a_ in Figure 7d, from which the bulk sensitivity is determined as 883 nm/RIU. The bulk sensitivity from the cross-correlations is smaller than that obtained directly from the spectra in which the Fabry–Pérot fringes are tracked (Figure 7a), because the Fabry–Pérot fringes are more sensitive than the underlying wide grating peak mostly tracked by the cross-correlation. However, tracking the cross-correlation is more robust, as the entire response curve is used, and there is no need to identify and track a specific feature.

**Figure 8:**
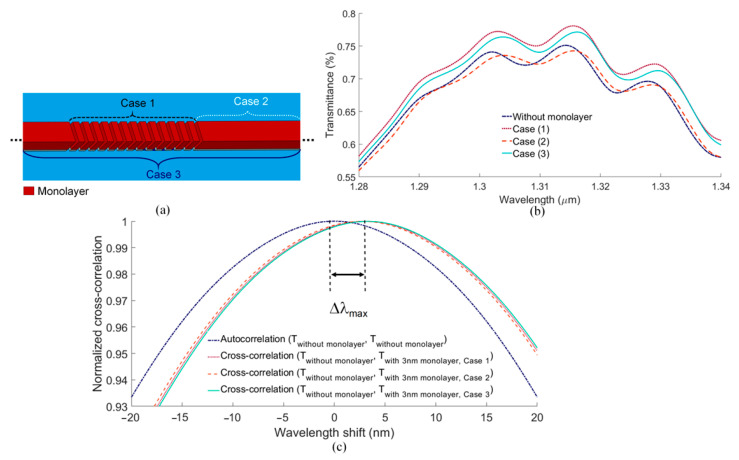
(**a**) Monolayer growth on different regions of the biosensor (shown for a small section of the structure). (**b**) Effect of 3 nm monolayer growth on the transmittance spectrum for all cases. (**c**) Calculated normalized autocorrelation of the transmittance without a monolayer and cross-correlations for a 3 nm monolayer in the three cases.

To evaluate the surface sensitivity of our proposed structure, we apply a 3 nm thick dielectric monolayer, representative of a monolayer of protein receptor molecules, on different regions of the sensor, as sketched in Figure 8a. Three separate cases of monolayer growth are considered: on the PSGCs only (Case 1), on the biosensing waveguide only (Case 2), and on the entire structure (Case 3). Although, in practice, the monolayer would grow following Case 3, i.e., on all exposed Au surfaces following bio-functionalization [41], considering different regions separately determines the contribution of each to the surface sensitivity. The RI of the buffer is set to 1.338, and the RI of the monolayer is set to 1.5, representative of proteins over a broad wavelength range.

Figure 8b plots the zoomed-in transmittance spectra computed for the three cases of monolayer growth. The growth of the monolayer in all cases shifts the transmittance. This means that both gratings and the waveguide contribute to the surface sensitivity of the device. We also calculate the normalized cross-correlation of the transmittance responses without and with the monolayer (for the three cases), as shown in Figure 8c. As observed, the results are almost the same for the different cases of monolayer growth. The surface sensitivity of the structure is taken as that for Case 3, which produces a shift of approximately 3.17 nm in the cross-correlation curves, yielding a surface sensitivity of approximately 1 nm/nm.

## 6. Discussion and Concluding Remarks

A comparison of our biosensor performance with other optical fiber end-facet (tip-based) biosensors in the literature is presented in Table 2. Such biosensors are generally based upon SPR [1,42,43,44] or localized SPR (LSPR) structures [45,46], Fano-resonance (FR) structures [47], Fabry–Pérot interferometers (FPIs) [48,49], or a grating-coupled FPI [13]. Most are based on single-mode fibers (SMFs), multi-mode fibers (MMFs), or photonic crystal fibers (PCFs) [33]. Wavelength interrogation in all cases is performed in reflection, except for three schemes [13,33,42]: one uses a DCSMF, but with a dielectric sensing structure on the end-facet [13], another uses two spectrometers and a splitter [42], and the other a free-space transmission setup [33]. Our design produces a high bulk sensitivity of approximately 880 nm/RIU, outperforming all of the end-facet biosensors reported in the literature, except for the design of [43] (Table 2).

Our proposed configuration exploits the main advantage of a DCSMF, i.e., enabling end-facet spectral interrogation using a fiber-based transmission setup (which is simpler than spectral interrogation in reflection). The configuration offers compelling prospects for remote real-time biosensing. The proposed configuration yields robust and experimentally realizable designs, e.g., using metal evaporation and FIB milling. Cross-correlation analysis is proposed as an approach to monitor spectral changes during sensing, which adds robustness to the measurements because the entire spectral response curve is used. The grating design approach based on the grating vector and modal analysis could be applied to design similar plasmonic grating couplers for other biophotonic applications. The proposed biosensor provides high bulk and surface sensitivities, although the latter could be further improved by enhancing the resonance between the two fiber cores or by nano-structuring the biosensing waveguide. The biosensor tip can be dipped in a vial containing the sensing sample, which eliminates the need for microfluidics and further simplifies the setup. Once packaged adequately, the biosensor could be inserted directly into a living organism for in vivo biosensing.

## Figures and Tables

**Table 1:** Design parameters of the proposed biosensor.

Parameter	Definition	Parameter Value
*θ*	slant angle	42.8°
Λ	grating pitch	971.67 nm (initial) 980.72 nm (scaled)
ff	fill factor	60%
w	grating width	583 nm (initial) 588.43 nm (scaled)
H_Cr_	chromium height	50 nm
H_Au_	gold height	300 nm
D_c_	core diameter	8 μm
L	grating length on the core	8 μm
L_off-b_	backward offset length	6 μm
L_off-f_	forward offset length	0
L_t_	total grating length (L + L_off-b_)	16 μm
D_inter_	intermediate distance between the cores	46.6 μm

**Table 2:** Comparison of optical fiber end-facet sensors with our proposed scheme.

Structure	S (nm/RIU)	RIU Range	Wavelength Range (nm)	Wavelength Interrogation Mode *	Data Type	Optical Fiber
SPR [1]	420	1.330–1.365	650–950	R	Experimental	MMF
SPR [42]	595	1.341–1.368	810–930	T/R	Experimental/Numerical	MMF
SPR [43]	2300	1–1.38	1250–1650	R	Experimental/Numerical	SMF
SPR [44]	294	1.32–1.38	700–1100	R	Experimental/Numerical	SMF
SPR/LSPR [33]	166.67–233.33	1–1.3	600–1100	T	Experimental/Numerical	PCF
LSPR [45]	125	1–1.4	1200–1550	R	Experimental/Numerical	SMF
LSPR [46]	755	1–1.5	800–1800	R	Experimental/Numerical	SMF
FR [47]	400	1.32–1.35	1220–1420	R	Experimental/Numerical	SMF
FPI [48]	5	1.31–1.47	1530–1570	R	Experimental/Numerical	SMF
FPI [49]	154	1.333–1.443	1260–1300	R	Experimental	SMF
Gratings/FPI [13]	50–100	1.33–1.52	1100–1500	T	Numerical	DCSMF
This work	883.2	1.33–1.39	1100–1500	T	Numerical	DCSMF

* R: Reflection; T: Transmission.

## Data Availability

Not applicable.

## Abbreviations

BMA	Boundary Mode Analysis
DCSMF	Dual-Core Single-Mode Optical Fiber
FEM	Finite Element Method
FIB	Focused Ion Beam Milling
FoM	Figure of Merit
FR	Fano-Resonance
FPIs	Fabry–Pérot Interferometers
FSR	Free Spectral Range
LSPR	Localized Surface Plasmon Resonance
LOF	Lab-on-a-Fiber
MMFs	Multi-Mode Fibers
PCFs	Photonic Crystal Fibers
PMLs	Perfectly Matched Layers
RI	Refractive Index
SMFs	Single-Mode Fibers
SPPs	Surface Plasmon Polaritons
SPR	Surface Plasmon Resonance
PSGCs	Plasmonic Slanted Grating Couplers
TM	Transverse Magnetic
